# Zenker’s diverticulum: Rotterdam experience

**DOI:** 10.1007/s00405-015-3825-0

**Published:** 2015-11-17

**Authors:** L. J. Visser, J. A. U. Hardillo, D. A. Monserez, M. H. Wieringa, R. J. Baatenburg de Jong

**Affiliations:** Department of Otorhinolaryngology, Head and Neck Surgery, Erasmus University Medical Center, ‘s-Gravendijkwal 230, 3015CE Rotterdam, The Netherlands

**Keywords:** Pharynx, Diverticulum, Dysphagia, Endoscopy, Zenker

## Abstract

Different surgical techniques exist for the treatment of Zenker’s diverticulum (ZD), of which minimally invasive techniques have become the standard. We reviewed our experience with management and treatment of ZD and sought to determine what type of treatment is most effective and efficient. We selected patients who underwent treatment for ZD between January 2004 and January 2014 at our tertiary referral center. All procedures were performed by ENT surgeons. The medical records were reviewed for pre- and intraoperative characteristics and follow-up. Of our 94 patients (58 male, 36 female), 75 underwent endoscopic cricopharyngeal myotomy (42 stapler, 33 laser) and 6 received treatment via transcervical approach. 13 interventions were aborted. Mean operating time was 49.0 min for stapler, 68.3 for laser and 124.0 for the transcervical approach. Its respective median post-operative admission durations were 2.0, 3.0 and 3.0 days. After the first treatment, of the 75 endoscopic procedures, 45 patients (23 stapler, 22 laser) had complete symptom resolution. In the transcervical group 4 (67 %) patients were symptom free and one patient died of complications. In the endoscopically treated patients, ten complications occurred, of which 8 G1 and 2 G2 (Clavien Dindo classification). In the transcervical group 2 complications occurred, 1 G3b and 1 G5. Both endoscopic techniques provide efficient management of Zenker’s diverticulum with the stapler-assisted modality providing a shorter surgery duration and hospital admission. Although there is no significant difference in terms of complications or recurrence rates for both endoscopic techniques, it seems that stapler patients are at higher risk of having a re-intervention and of having more severe complications.

## Introduction

Zenker’s diverticulum (ZD) is a hypopharyngeal pouch caused by herniation of all muscular layers through an area of weakness between the transverse fibers of the cricopharyngeus muscle and the oblique fibers of the lower inferior constrictor muscle, called Killian’s dehiscence or the triangle of Killian.

ZD is uncommon, with an estimated incidence of 2 per 100,000 per year in the United Kingdom [1]. However, it is the most common type of esophageal diverticulum [2]. Patients regularly present with symptoms of dysphagia, regurgitation, aspiration, chronic cough and weight loss [3]. The diagnosis is more frequent in elderly males and has a peak incidence between the ages of 70 and 90 [4].

Though first identified in 1769 by Abraham Ludlow [5], it was not until the nineteenth century that Zenker and von Ziemssen [5] termed and fully described the diverticulum. Harris Mosher was in 1917 the first to describe treatment for ZD through endoscopic diverticulotomy [4], in which he used punch forceps to take down the intermediate septum. However, the risk of complications was too high and it was not until the reintroduction of this approach in 1960 by Dohlman and Mattsson [6], reporting good results and permissible morbidity, that it finally gained acceptance.

Although several theories existed regarding the pathogenesis of the pouch, also termed cricopharyngeal or pharyngoesophageal diverticulum, it is now generally thought the pouch is due to a combination of increased hypopharyngeal pressure, brought forth during deglutition [7] and dysfunction of the cricopharyngeal muscle [8].

The only known curative treatment for ZD is surgery [9], aiming at complete and sustaining dissolution of symptoms in combination with early commencement of oral intake and short hospital stay without complications. It appears that the most essential part of surgical treatment is the parting of the cricopharyngeal muscle [8]. Treatment of ZD at the department of Otorhinolaryngology, ErasmusMC is either through a transoral (CO_2_ laser or stapler) approach or by open surgery (transcervical cricopharyngeal myotomy, either alone or in combination with diverticulectomy or diverticulopexy).

In this paper, we review our experience with the management and treatment of Zenker’s diverticulum. We compare the frequencies of the various treatment types and their associated complications, admission durations and how often patients have a recurrence. We sought to analyze and determine what type of treatment is most effective.

## Materials and methods

Institutional review board permission was obtained to retrospectively analyze records of patients who underwent surgical management of ZD at the Department of Otorhinolaryngology, Head and Neck Surgery (ORL), Erasmus Medical Center. All patients from this department who received treatment for Zenker’s Diverticulum, between 01-01-2004 and 01-01-2014 were included in this study. Patients were recognized by operation code from a database. Patient files were then individually reviewed to obtain data and subsequently anonymized, using a code list of study numbers and patient numbers. Patients who were previously treated for ZD at another hospital (1), patients with insufficient data (5) and patients with malignant hypopharyngeal/esophageal tumor were excluded (2). Patients who were operated on with surgical methods that are no longer in use or methods that are not used frequently (five or less per 10 years), were also excluded (one coagulation, five stapler combined with laser).

Data on preoperative analysis, medical history, head and neck examination and barium swallow radiography or video fluoroscopy were collected from all patients. The latter not only confirmed diagnosis but also revealed the size and location of the diverticulum and the patients’ swallowing functions.

### Preoperative assessment

Preoperative planning was performed in the outpatient clinic and the surgical approaches are discussed with the patient. Surgical treatment is either by a transcervical approach (TA) or by transoral endoscopic approach, which can be subdivided in an endoscopic laser-assisted diverticulotomy (ELAD) or an endoscopic stapler-assisted diverticulotomy (ESAD). In the case of ELAD a CO_2 _laser is used. Selection of surgical technique depends on various factors such as the extent of accessibility of the diverticulum via the oral cavity, length of the diverticulum, presence of comorbidities, patients’ preference and surgeons’ preference.

 The feasibility of an endoscopic procedure is mainly assessed, based on the following clinical criteria (protrusion of upper teeth, recessed mandible, degree of mouth opening and mobility of cervical spine, and radiological findings (size of diverticulum and relation with posterior esophageal wall).

### Surgical procedures and postoperative course

All transoral endoscopic procedures are performed using an endotracheal (ET)-tube under general anesthesia and in the case of dentate patients, a dental guard is used. Prior to actual surgery, the mucosa of the diverticulum and the upper part of the esophagus are inspected for suspicious abnormalities. If suspicious lesions are found, they are biopsied and sent to a pathologist. For the ELAD technique, the Dohlman laryngoscope is introduced to expose the septum (cricopharyngeal muscle) between the esophagus and the diverticulum. The shorter lip is positioned in the diverticulum and the longer one in the esophagus. The exposed septum (cricopharyngeal muscle) is then divided by the continuous wave of the CO_2_ laser. Subsequently, a nasogastric feeding tube is inserted under direct vision.

For the ESAD technique, the Overbeek laryngoscope (which is larger in diameter than the Dohlman laryngoscope) is used to expose the common wall or septum. The endosurgical stapler (Endo GIA™, Covidien) is inserted through the laryngoscope. During firing, the Endogia cuts through the lumen and simultaneously staples the mucosa, thereby reducing possible bleeding. A small remnant of the septum usually remains due to the design of the stapling device. No nasogastric tube is required.

The transcervical approach (TA) consists of a left-sided cervical incision along the anterior body of the sternocleidomastoid muscle. Subsequently, the underlying structures are dissected to locate the diverticulum and the cricopharyngeal (CP) muscle. The identification of the diverticulum and the CP muscle is facilitated by inserting a probe or an esophageal bougies transorally. The cricopharyngeal myotomy is performed until the longitudinal muscle fibers of the esophagus are reached.

All patients were postoperatively observed for signs of thoracic pain or pain between the scapula, fever, subcutaneous emphysema and dyspnea. If patients had any of these complaints, a chest X-ray was made to rule out mediastinitis. Patients whose diverticula were stapled without complications or patients who received external treatment were immediately started on oral clear fluids. If uneventful, a soft meal was introduced on the evening of the surgery. Patients who received ELAD were kept from oral fluids or food for at least 24 h and were fed via the nasogastric tube. After 24 h patients commenced intake with water, after which the diet was gradually expanded, provided that observations remained satisfactory. Follow up or control barium swallow is not done unless there is recurrence of symptoms similar to that prior to surgery during regular follow up.

### Data analysis

Patient data were collected from the patients’ medical files, operative charts and radiological findings. This data included: date of birth, gender, date of visit to outpatient clinic, medical history, pre- and post-operative symptoms, characteristics of the diverticulum, type of surgery, duration of surgery, admission duration, complications, whether patients had a recurrence and if applicable, the reason for referral to other specialties. Surgical success was recorded when patients had no recurrence of complaints. A patient was considered as having full resolution of symptoms if they no longer reported any symptoms they had previous to treatment during follow up. Patients who had partial symptom resolution, at the first visit following surgery, who subsequently became asymptomatic, were recorded as having full resolution of symptoms. In this study we defined recurrence as the reappearance of the same symptoms before the initial operation and not necessarily the presence of the need for reintervention/reoperation.

Statistical analysis was performed using SPSS version 21.0. To compare the surgical interventions, a variety of statistical tests were used on the outcome variables. In order to compare the duration of the surgery, we used the two-sample *t* test. For comparison between post-operative and total admission duration, we used the Mann–Whitney *U* test. Finally, to compare between the rates of recurrence of symptoms and re-intervention we used the Chi-squared test. Values of *p* < 0.05 were considered to indicate statistical significance.

## Results

### Patients

Between January 2004 and January 2014, 124 patients underwent surgery for Zenker’s Diverticulum at the Erasmus MC, Department of Otorhinolaryngology, Head and Neck Surgery. 24 patients were excluded from further investigation because of: incomplete medical charts (16), previous surgery in other medical centers (5) or the presence of malignant oropharyngeal or hypopharyngeal cancer (3). Another six patients were excluded from analysis since they underwent techniques that are not frequently used in our center i.e, stapler combined with laser in five patients and myotomy using a monopolar diathermy in a single patient. Consequently, 94 patients were included in this retrospective study, whose characteristics are shown in Table 1. Radiological images of the diverticula were mostly done in the referring clinics and the differences in the measurements which are mainly technical in nature precluded a meaningful comparison of the sizes of the diverticula.

**Fig. 1:**
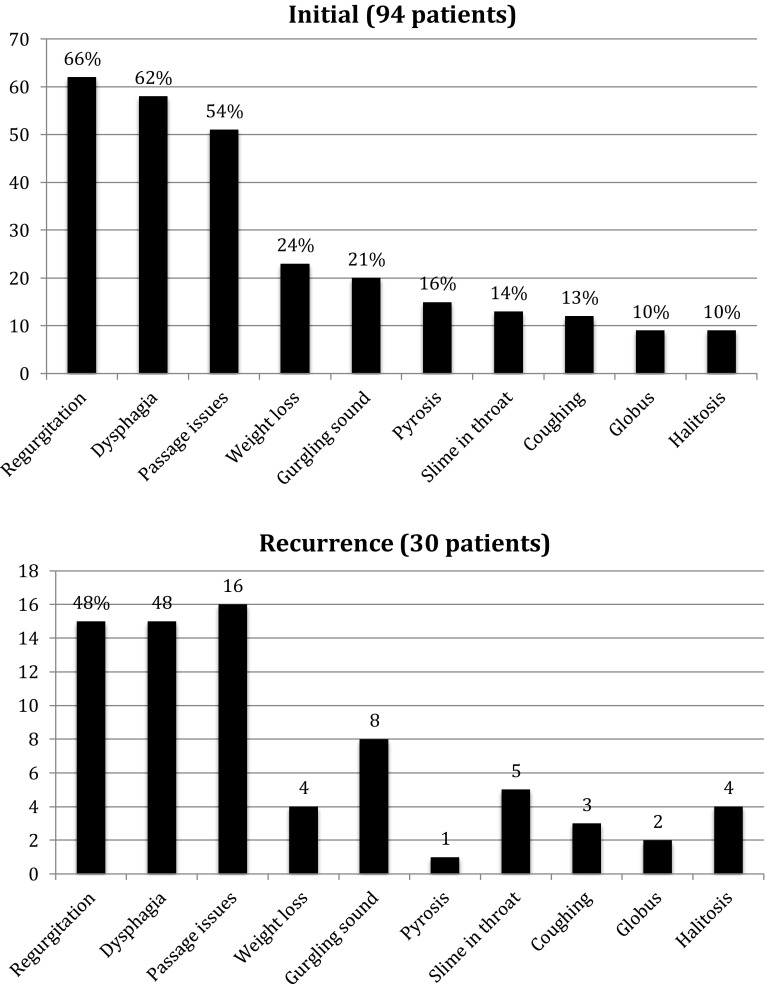
Symptoms

**Table 1:** Population characteristics

	ESAD	ELAD	TA	Aborted	Total
Sex					
Total	42	33	6	13	94
Male (%)	25 (60)	21 (64)	3 (50)	9 (69)	58 (62)
Female (%)	17 (40)	12 (36)	3 (50)	4 (31)	36 (38)
Age					
Mean	72	69	68	62	69.4
SD	11.5	9.6	15.2	8.4	11
Range	44–96	42–85	43–80	46–74	42–96

**Table 2:** Intervention characteristics

	ESAD (*n* = 42)	ELAD (*n* = 33)	p*	TA (*n* = 6)	Total (81)
Surgery duration (min)			0.00		
Mean	49.0	68.3		124	62.3
SD	17.9	21.6		34.1	28.7
Post-op admission duration (days)			0.01		
Median	2.0			3.0	3.0
Range	0–44			2–14	0–44
IQR	1–4			2.75–8.75	2–4
Total admission duration (days)			0.03		
Median	4.0	5.0		4.5	5.0
Range	2–45	2–13		3–15	2–45
IQR	3–6	4.5–6		3.75–9.75	4–6
Initial recurrence (%)	19 (45 %)	11 (34 %)**	0.35	0	30 (37 %)
Re-operated by ORL	7	3			10
Referral	5	4			9
No re-intervention	7	4			11
Complications (%)	6 (14)	4 (12)	0.75	2 (33)	12 (15)
Minor (%)	4 (10) G1^a^	4 G1^a^		1 (17) G1^a^	9 (11)
Major (%)	2 (5) G2^a^			1 (17) G5^a^	3 (4)

* *p* value for comparison between ESAD and ELAD

** Corrected for missing data

*** IQR is the difference between the upper and lower quartiles

^a^Classification of surgical complications according to Clavien and Dindo [16]

### Surgery and frequency (Table 2)

The distribution of the different types of surgery was as follows: 42 ESAD, 33 ELAD and 6 TA. 13 interventions had to be aborted, because of a non-accessible diverticulum (8), a common wall that proved to be too small to be divided (3) and two more, which were not further specified. In six of these cases both endoscopic modalities were tried, in three ESAD, in another three ELAD and one wasn’t specified. Of the six patients operated via TA, five patients received a myotomy of the cricopharyngeal muscle, one had the complete diverticulum removed by a stapling device (diverticulectomy).

### Recurrences and re-interventions (Table 2)

The number of recurrences for the various techniques after the first intervention was as follows: ESAD 19 (45 %), ELAD 11 (33 %) and TA 0. There were no statistically significant differences between the number of recurrences found in the ESAD and ELAD group (*p* = 0.35). These recurrences resulted in 12 (29 %), 7 (21 %) and 0 re-interventions, respectively.

For the 12 re-interventions in the ESAD group, seven patients were re-operated on by the Department of Otorhinolaryngology (ORL), four patients were referred to the Department of Gastroenterology and Hepatology (GEH) and one to the Department of Surgery, all at our center. The reasons for the referrals to GEH were; the remaining septum was too short (1) or too distal (1) for further excision using our transoral endoscopic techniques, the longer waiting list at ORL (1), or unknown (1). The reason for referral to the Department of Surgery was the length of the waiting list as well.

The remaining seven recurrences treated with ESAD, were not re-operated because; the complaints were too mild for intervention (3), severe comorbidity (2), patient’s wish not to intervene (1) and death due to other causes (1). The seven re-interventions at the department of ORL were all done with ELAD of which three resulted in a complete resolution of symptoms and four resulted in a second recurrence. Of these 4 s recurrences two were referred to GEH, one patient was not operated on because of the complaints being too mild and one because of patient’s wish.

For the 11 recurrences of symptoms in the ELAD group, three patients were re-operated on by ORL. Four patients were referred to GEH because of a non-accessible diverticulum (2) or for unknown reasons (2). Four patients received no surgery because the complaints were too mild (2), because of the patients’ wish (1) or for unknown reasons (1). The three re-interventions at the department of ORL resulted in 2 ELAD treatments and one intervention being cancelled. The two ELAD re-interventions resulted in one patient being symptom-free and one patient with a second recurrence. The patient with the second recurrence did not receive a third operation because the complaints were considered to be too mild. The cancellation was because the diverticulum was not accessible and therefore the patient was referred to GEH (Table 3).

**Table 3:** Clavien Dindo classification of surgical complications

Grade	Definition
Grade I	Any deviation from the normal postoperative course without the need for pharmacological treatment or surgical, endoscopic and radiological interventions. Allowed therapeutic regimens are: drug as antiemetics, antipyretics, analgetics, diuretics, electrolytes and physiotherapy. This grade also includes wound infections opened at the bedside
Grade II	Requiring pharmacological treatment with drugs other than such allowed for grade I complications. Blood transfusions and total parenteral nutritions are also included
Grade III	Requiring surgical, endoscopic or radiological intervention
IIIa	Intervention not under general anesthesia
IIIb	Intervention under general anesthesia
Grade IV	Life threatening complications (including CNS complications)* requiring IC/ICU management
IVa	Single organ dysfunction (including dialysis)
IVb	Multiorgan dysfunction
Grade V	Death of patient
Suffix “d”	If the patient suffers from a complication at the time of discharge the suffix “d” (for disability) is added to the respective grade of complication. This label indicates the need for a follow-up to fully evaluate the complication

A single patient in the group whom were treated via the transcervical approach had complaints that seemed like symptoms of Zenker’s diverticulum. However, they turned out not to be related to a diverticulum but to achalasia of the lower esophagus and gastroesophageal reflux. The patient was then referred to the Department of Gastroenterology.

### Duration of surgery (Table 2)

The overall duration of the surgeries varied from 25 to 168 min, with a mean duration of 62 min (SD 29). The ESAD had a mean duration of 49 min (SD 18). The ELAD intervention took significantly longer (*p* < 0.01), where we found a mean duration of 68 min (SD 22). The TA intervention had a mean duration of 124 min (SD 34).

### Duration of admission (Table 2)

The overall total admission duration of ESAD, ELAD and TA varied from 2 to 45 days with an overall median of 5 days (IQR 4–6). The post-operation admission duration varied from 0 to 44 days with an overall median of 3 days (IQR 2–4). For the ESAD patients we found a median total admission duration of 4 days (IQR 3–6) and a median post-operation admission duration of 2 days (IQR 3–4). In the ELAD group, we found a median total admission duration of 5 days (IQR 4.5–6) and a median post-operation admission duration of 3 days (IQR 3–4). In both the total admission duration and the post-operation admission duration we found a statistically significant difference between the ESAD and the ELAD group (respectively, *p* = 0.033 and *p* = 0.010). Finally, the external approach patients had a median total admission duration of 4.5 days (IQR 3.75–9.75) and a median post-operation admission duration of 3 days (IQR 2.75–8.75).

### Symptoms (Fig. 1)

The most frequent preoperative symptoms were regurgitation, dysphagia and the feeling of food getting stuck. Furthermore, patients complained of weight loss, a gurgling sound after a meal and pyrosis. A smaller group of patients had a surplus of mucus in their throats, were coughing, had the feeling that there was something stuck in their throat or suffered from halitosis.

Postoperatively, the symptoms most frequently reported by patients who had a recurrence of symptoms were the same, however, in different order. If patients had a recurrence of symptoms, the most frequent symptoms were feeling of food not passing well, dysphagia and regurgitation. Additionally, patients reported a gurgling sound, a surplus of mucus in their throats and halitosis.

### Complications (Table 2)

A total of 12 complications have been registered in the medical charts and surgical records. In the ESAD group, 6/42 (14.3 %) patients had complications, four of which had a damaged epithelium or muscular wall of the pharynx or diverticulum. Two of these resulted in mediastinitis and antibiotics-use-related hepatic dysfunction (both were successfully treated with amoxicillin with clavulanic acid with or without gentamicin) and one resulted in hematemesis. Skin lesions due to the positioning of a patient during surgery, subcutaneous emphysema and a fever with an unknown origin were seen in one case. One additional patient suffered from subcutaneous emphysema without fever.

In the laser intervention group we found a total of 4/33 (12 %) patients with complications. One patient had a damaged tooth due to insertion of a diverticuloscope. Another patient had a damaged posterior pharynx wall, which did not result in mediastinitis. A third patient had a temporarily paralyzed vocal cord and one patient, using anticoagulants, suffered from a minor bleeding which was treated conservatively

Two of the six (33 %) patients who were treated via the external approach had complications. The first patient suffered from dyspnea and laryngeal swelling 1-day post surgery, which required an emergency tracheotomy further complicated with bleeding. She suffered severe brain damage due to hypoxia, which subsequently resulted in death. The second patient had a minor bleeding at the surgical area which spontaneously stopped.

## Discussion

Discussion remains as to what technique is to be preferred in managing Zenker’s diverticulum. Chang [10] in his review concluded that stapler-assisted diverticulotomy was favorable over laser and Verhaegen [11] agreed with this in 2011. The reviews of Dzeletovic [12] in 2012 and Yuan et al. [13] in 2013 however stated that every treatment type has its advantages and disadvantages and therefore treatment should be tailored to the patients’ characteristics.

In our study we found the stapler-assisted diverticulotomy to be slightly favored over the laser-assisted treatment in terms of frequency of use. This is expected since ESAD has been associated with shorter surgery duration, faster onset of oral intake and shorter hospital admission. We also found them both to be heavily favored over TA. This, however, was also to be expected, since it has been stated and proven in studies [7, 10, 14] that the transcervical approach is less cost-effective and less safe in comparison to endoscopic treatments. However, despite the proven benefits of ESAD and ELAD over TA, the anatomical accessibility of a diverticulum using an endoscope might be limited and treatment may be impossible in the case of a small diverticulum. The open approach offers a better visualization of the diverticulum and the CP muscle and thus for sufficient myotomy of the cricopharyngeal muscle. In our study we consider the number of patients treated via TA too small to make an accurate comparison with the other treatments.

### Re-interventions and recurrences

Reasons for recurrences of symptoms in patients treated for ZD could be incomplete division, stenosis or scar formation at the cleavage site of the bridge (CO_2_ laser) or an incomplete division of the common wall as a result of the nonfunctional end of the stapler blade. Adams et al. [15] stated that because of this, endoscopic diverticulotomy with stapler is not advisable if the pouch is smaller than 3 cm deep. As stated before, it has been shown that the endoscopic modalities are safer and more cost-effective compared to TA. However, there are disadvantages associated with these minimally invasive strategies, since it has been found that higher recurrence rates are associated with the endoscopic approaches compared with an open technique [10].

At first glance, the percentage of patients in our study who had a recurrence of symptoms is relatively high compared with the rates in the reviews by Chang, Dzeletovic, but low compared to Verhaegen [11]. However, we should take into account that these reviews did not clearly define what was meant by recurrence. Chang for example stated that different studies apply different definitions as to whether a case is considered a recurrence or not. Some studies define ‘recurrence’ as a re-intervention, others merely as the recurrence of symptoms. Furthermore, they state that in some studies, patients in certain cases underwent repeated endoscopic divisions of the common wall, until they were symptom free or improved, while processing the treatments as one intervention. Therefore, it is very difficult to compare recurrence rates from different studies with our own. In addition, the relatively high recurrence of symptoms rates could also be the result of a cautious approach, as reflected by the relatively low rate of major complications.

### Duration of surgery and admission

 Comparing to the study done by Verhaegen et al. [11], we found a comparable median postoperative hospital stay for the ESAD group (2 days), but less days of hospitalization for the ELAD patients (five compared to 3 days). When comparing the latter group of patients to Chang et al., who are using the mean instead of median, we can still see that we do relatively well on post-operative hospitalization for ELAD patients. The ESAD difference is less evident. Yuan et al. do not specify whether the hospital stay is the total admission or the post-operative admission and therefore not comparable. Dzeletovic reported no numbers on hospital stay.

The mean operating times in the review of Chang are lower than ours, (ESAD 27.2 compared to our mean 49.0). ELAD is not reported. The results of Yuan also indicate that we have relatively long durations of surgery. Verhaegen and Dzeletovic reported no numbers on operating time.

### Complications

A total of 15 % of the patients had complications associated with the treatment they received. Though the majority (12 %) of these patients had minor complications (Grade 1 complications following the Clavien and Dindo classification) such as minor bleedings at wound area. There were three cases that we consider major complications (two Grade 2 and one grade 5 complications according to the Clavien and Dindo classification). The most severe was a patient who was treated via TA. The first day postoperatively, the patient developed severe dyspnea. Despite tracheotomy, this resulted in hypoxia, severe brain damage and eventually to death. The other two complications, both in the ESAD group, consisted of two patients who developed mediastinitis due to a damaged muscular wall of the diverticulum and subsequently antibiotics-related liver damage. The four Grade 1 complications in the ESAD group, all of which were superficial lesions of the hypopharyngeal/esophageal mucosa, probably occurred during the insertion and manipulation of the Overbeek laryngoscope that is used in combination with the stapling device. As stated before, this laryngoscope is of a greater diameter than the one used for ELAD.

Chang et al. [10] did not clearly describe what was meant by complication, but merely called it ‘significant complications’. They reported an average complication rate of 7.4 % in the laser group and 2.6 % in the stapler-group. Verhaegen reports two minor complications in the CO_2 _laser group and none in the stapler group. Dzeletovic reports a median of 3 % of major complications, which is comparable to our study. Yuan found an overall complication rate of 8.7 %, mostly complications we would consider major.

In our study, we saw a number of patients with minor complications such as a temporarily paralyzed vocal cord or a damaged posterior pharynx wall. Whether this is to be considered a ‘significant complication’ is questionable, since it did not have any further consequence.

If we take a look at our rate for the major complications we reported, we find it relatively low compared to the rate reported by Chang. However, as we stated before with recurrence rates—we should take into account that it remains difficult to compare results of reviews with our present study, for reasons of semantics.

### Limitations and bias

The main limitation is the small number of patients due to the rarity of the disease. Although we chose a relatively wide time frame, we still yielded few patients. Another limitation is the quality of the follow-up: there are no standardized questionnaires or protocols to accurately assess a patient’s improvement after treatment. This would certainly improve the loss of quality typically associated with retrospective studies.

### Variables

Both total admission duration and post operation admission duration are clearly mentioned in our results. Since ZD is a condition more often found in elderly patients than younger people, patients have more comorbidity. This results sometimes in an early pre-operative admission, in order to monitor the patients’ overall condition. If we would only analyze the total admission duration instead of the post-operative admission and would associate this with the various treatment options, the numbers would distort the actual data.

### Summary and conclusion

Although it seems that all three approaches have advantages and disadvantages, the endoscopic modalities remain the treatments of choice, for research has proven these methods to be favored over TA. However, once the choice for endoscopic treatment has been made, there remains the choice whether to use the laser or the stapler. This consideration is based on both anatomical accessibility and surgeons’ preference.

Comparing the stapler with the laser-assisted surgery; our data show that the duration of surgery and the admission duration (both total and post-operative) are significantly lower in patients treated with stapler compared to patients treated with laser, thus possibly contributing to lower costs. In addition, the stapler-assisted technique enables an early commencement of oral intake, thereby lowering chances of complications due to malnutrition. However, according to the Clavien Dindo classification of surgical complications, we see a total of two G2 complications in the ESAD group, whereas the ELAD group has none. And, as stated before, the design of the stapler decreases the possible gain a surgeon can achieve in dividing the common wall, thereby directly increasing the risk of a recurrence or incomplete symptom relief.

To conclude, we can state that both endoscopic techniques provide efficient management of Zenker’s Diverticulum with ESAD providing a shorter surgery duration and hospital admission. Although there is no significant difference in terms of complications or recurrence rates for both endoscopic techniques, it seems that ESAD patients are at higher risk of having a re-intervention and of having more severe complications. The jury is still out on this one.
